# Microbial‐ and host immune cell‐derived extracellular vesicles in the pathogenesis and therapy of periodontitis: A narrative review

**DOI:** 10.1111/jre.13283

**Published:** 2024-05-17

**Authors:** Jenny Wang, Chun Liu, Jason Cutler, Sašo Ivanovski, Ryan SB Lee, Pingping Han

**Affiliations:** ^1^ School of Dentistry, Center for Oral‐facial Regeneration, Rehabilitation and Reconstruction (COR3), Epigenetics Nanodiagnostic and Therapeutic Group The University of Queensland Brisbane Queensland Australia; ^2^ School of Dentistry The University of Queensland Brisbane Queensland Australia

**Keywords:** extracellular vesicles, immune cells derived EVs, inflammatory response, microbial EVs, pathogenesis, periodontitis, therapy

## Abstract

Periodontitis is a chronic inflammatory disease caused by dysbiotic biofilms and destructive host immune responses. Extracellular vesicles (EVs) are circulating nanoparticles released by microbes and host cells involved in cell‐to‐cell communication, found in body biofluids, such as saliva and gingival crevicular fluid (GCF). EVs are mainly involved in cell‐to‐cell communication, and may hold promise for diagnostic and therapeutic purposes. Periodontal research has examined the potential involvement of bacterial‐ and host‐cell‐derived EVs in disease pathogenesis, diagnosis, and therapy, but data remains scarce on immune cell‐ or microbial‐derived EVs. In this narrative review, we first provide an overview of the role of microbial and host‐derived EVs on disease pathogenesis. Recent studies reveal that *Porphyromonas gingivalis* and *Aggregatibacter actinomycetemcomitans*‐derived outer membrane vesicles (OMVs) can activate inflammatory cytokine release in host cells, while M1 macrophage EVs may contribute to bone loss. Additionally, we summarised current in vitro and pre‐clinical research on the utilisation of immune cell and microbial‐derived EVs as potential therapeutic tools in the context of periodontal treatment. Studies indicate that EVs from M2 macrophages and dendritic cells promote bone regeneration in animal models. While bacterial EVs remain underexplored for periodontal therapy, preliminary research suggests that *P. gingivalis OMVs* hold promise as vaccine candidates. Finally, we acknowledge the current limitations present in the field of translating immune cell derived EVs and microbial derived EVs in periodontology. It is concluded that microbial and host immune cell‐derived EVs have a role in periodontitis pathogenesis and hence may be useful for studying disease pathophysiology, and as diagnostic and treatment monitoring biomarkers.

## 1 INTRODUCTION

Periodontitis is a chronic inflammatory disease associated with dysbiotic polymicrobial biofilms, affecting up to 62% of the dentate adult population worldwide, with over 23.6% having severe forms of the disease.^1^ The complex crosstalk between the polymicrobial biofilm and the host immune system plays a crucial role in the pathogenesis of periodontitis,^2^, ^3^, ^4^, ^5^ highlighting the importance of exploring the secretome derived from both sources, specifically extracellular vesicles (EVs).^6^

EVs are lipid‐bound vesicles secreted by both host and microbial cells into biological fluids ranging in size from 20 nm to 5 μm.^7^ The cargo of EVs consists of biological molecules from their parent cells, including mRNAs, miRNAs, DNA, circular RNAs, lipids and proteins.^8^ Due to their double membrane structures, EVs are protected against enzyme degradation, enhancing their bioavailability for therapeutic targeting and detection in diagnostic tests.^9^, ^10^ While host‐derived EVs have been widely explored, bacteria derived EVs (BEVs), ie Gram‐negative bacteria derived BEVs (namely outer membrane vesicles‐OMVs), are limited in periodontology. In this review, we will use the term “EVs” as a general designation for all EVs, while specifying “BEVs” to represent bacterial EVs, and referring to “host‐EVs” as EVs derived from host cells in a broader sense.

In periodontology, current EV studies have centred around host derived small EVs as potential diagnosis tools,^11^, ^12^ or OMVs produced by in vitro culture of a single periodontal pathogen, or EVs derived from dental mesenchymal stem cells.^12^, ^13^, ^14^, ^15^, ^16^, ^17^, ^18^, ^19^, ^20^ Although immune cells and microbiota are crucial in the periodontal microenvironment,^21^, ^22^ little is known about the EVs derived from these cells in this context.

In this narrative review, our objective is to present an overview of the potential role played by microbial EVs and host immune cell derived EVs in the pathogenesis of periodontitis, underscore the prospects of utilising immune cell EVs in diagnosis and therapy and acknowledge the existing limitations within the realm of EV research.

## 2 BACTERIAL AND IMMUNE CELL DERIVED EVS IN THE PATHOGENESIS OF PERIODONTITIS

The pathogenesis of periodontitis has been described as a disproportionate and dysregulated immune response to a dysbiotic biofilm.^23^, ^24^ Disease and subsequent tissue destruction occur when the host response, potentially modified by environmental and systemic risk factors such as smoking and diabetes, is unable to contain the microbial dysbiosis and a destructive inflammatory response occurs.^25^ Gram‐negative bacteria in periodontal pockets such as *Porphyromonas gingivalis* (*P. gingivalis*), *Treponema denticola* (*T. denticola*), *Tannerella forsythia* (*T. forsythia*), *Actinomyces reticulata* (*A. reticulata*), *Fusobacterium nucleatum* (*F. nucleatum*) and *Prevotella intermedia* (*P. intermedia*) are positively associated with the progression of periodontitis.^26^, ^27^, ^28^ Therefore, investigations into microbial and host immune cells and their products, such as EVs, are crucial for a deeper understanding of periodontitis pathogenesis and potential therapeutic interventions.

This review does not aim to cover all EVs from every cell type, but briefly discuss single species periodontal bacterial cell derived EVs due to their established role in disease pathogensis.

### 2.1 Overview of extracellular vesicles in the periodontal microenvironment

**FIGURE 1:**
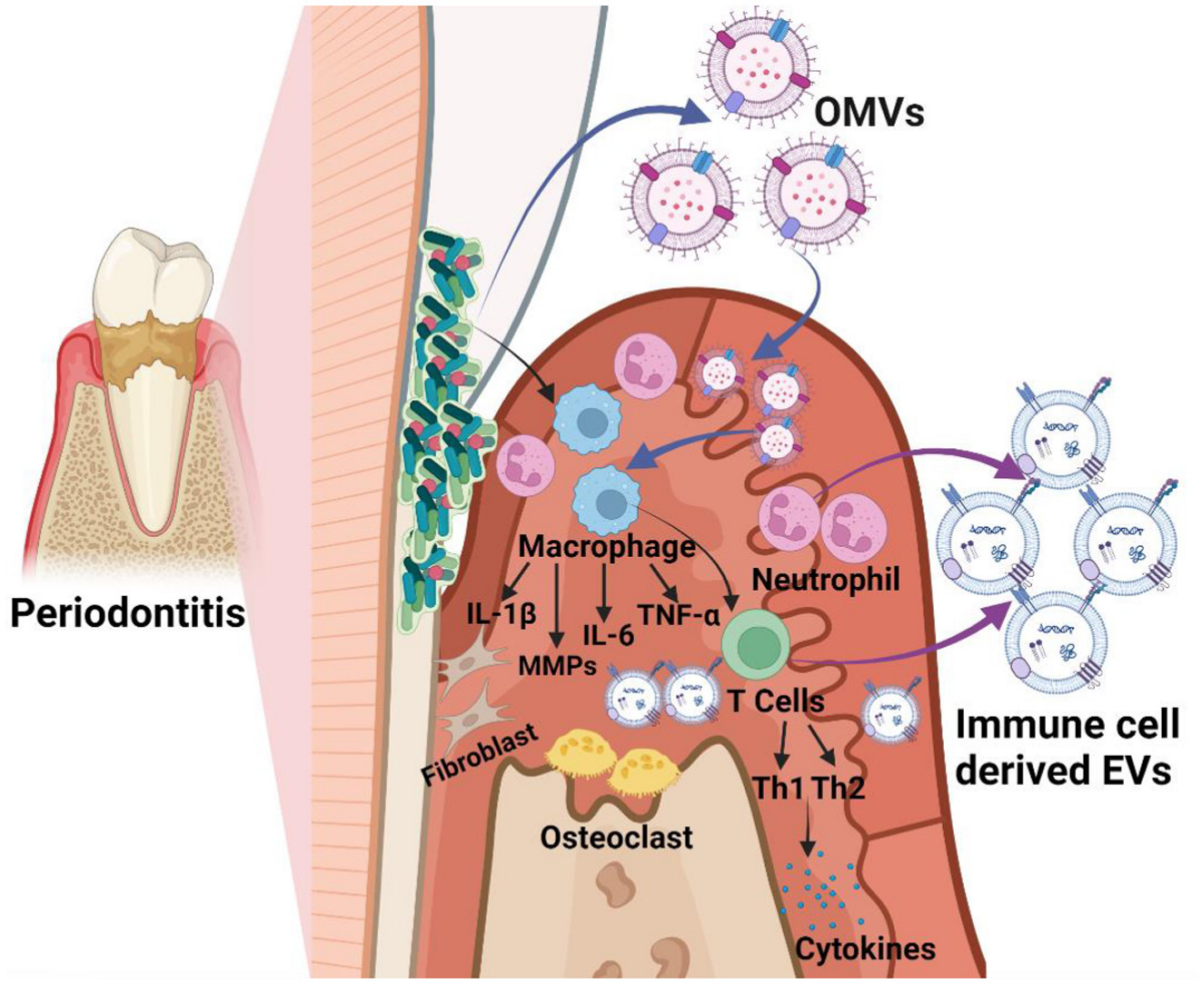
The potential roles of host‐derived EVs and bacteria‐derived OMVs in periodontal disease pathogenesis. Bacterial OMVs can enter the periodontal tissues via sites of injury, induce a reaction of pattern recognition receptors (PRRs) in host cells, activate immune cells and secretion of both pro‐ and anti‐inflammatory cytokines. These OMVs can also carry packages containing lipopolysaccharide (LPS) and other virulence factors that can potentiate disease via mechanisms such as recruitment of inflammatory mediators' interleukin 1β (IL‐1β), IL‐6 and tumour necrosis factor‐alpha (TNF‐α). Activation and recruitment of immune cells such as neutrophils, macrophages and antigen‐presenting cells occurs and EVs derived from these immune cells can in turn influence the differentiation and behaviour of other immune cells. Depending on the stimulus and the local environment, EVs can either aid in the resolution of the inflammation or perpetuate chronic inflammation with engagement elements of adaptive immunity such as T cells, T helper cells (Th1, Th2) and subsequent tissue destruction with the recruitment of pro‐inflammatory factors and increasing activity of matrix metalloproteinases (MMPs) and osteoclasts. OMVs: outer membrane vesicles; TH1, T helper 1; TH2, T helper 2.

In the periodontium, EVs can be secreted by a range of human, animal, bacterial and plant cells including immune cells such as macrophages, neutrophils and antigen‐presenting cells.^13^ EVs found in oral biofluids such as saliva and gingival crevicular fluid (GCF), may stem from cells of the periodontium, i.e., periodontal ligament and epithelial cells, bacteria, or immune cells (Figure 1). Recent evidence suggests that EVs from host cells, particularly those from inflamed or LPS and biofilm stimulated periodontal ligmanet fibroblast and gingival epithelial cells, negatively affect the host's immune response, angiogenesis and tissue integrity.^16^, ^29^ Bacterial‐derived EVs are known to carry virulence factors such as LPS, exacerbating disease.^16^ Despite periodontal disease involving chro nic inflammation and both innate and adaptive immunity, specific immune cell derived EVs have not been extensively researched in it is pathogensis. These EVs could influence the proportion and differentiation of immune cells and their response or tolerance to bacterial stimulus.^30^, ^31^ Therefore, it is not surprising there is growing interest in the use of EVs in oral biofluids to better understand periodontitis with goals of formulating clinically applicable diagnostic and therapeutic approaches.

### 2.2 Gram‐negative bacterial outer membrane vesicles in periodontitis

Outer membrane vesicles (OMVs) from gram negative bacteria have been extensively studied,^22^ while gram‐positive bacteria derived membrane vesicles (MVs) remain largely unexplored in periodontitis. OMVs are double‐layered membrane nanospherical structures with sizes ranging from 20 to 400 nm, secreted by gram‐negative bacteria.^22^ These vesicles mainly consist of an asymmetric lipid bilayer, with outer leaflets bearing numerous lipopolysaccharides (LPS).^13^ OMVs produced by periodontal pathogens such as *P.gingivalis* play a pivotal role in triggering and accelerating the progression of periodontal disease by delivering various virulence factors (e.g. outer membrane proteins, LPS, and lipids) to host cells (Figure 1).^32^ These OMVs trigger pattern recoginition receptors (PRRs) in gingival epithelial cells, leading to the secretion of pro inflammatory and anti inflammatory cytokines.^16^ This activation subsequently cascades to involve key immune effectors such as neutrophils, T and B lymphocytes, and osteoclasts.^16^ Through engaging in interactions with both other bacteria and host cells, OMVs serve to enhance the survival and evasion strategies of periodontal pathogens, intensify the inflammatory response, and contribute to periodontal tissue destruction.^13^, ^16^

Liu et al. confirmed that OMVs naturally secreted by *Fusobacterium nucleatum*, a species believed to be central to biofilm colonisation, carry many self‐transport proteins involved in pathogenic mechanisms such as induction of lymphocyte apoptosis and promotion of immune evasion.^33^, ^34^ OMVs from *Porphyromonas gingivalis*, a pathogen widely associated with periodontitis, have been found to contain only outer membrane and periplasmic proteins. These OMVs carry cargo rich in virulence factors which may potentiate an additional secretion pathway for toxicity and nutrient acquisition.^35^, ^36^ Compared to intact *P. gingivalis* cells, *P. gingivalis* OMVs show significantly higher invasive activity and higher expression of cytokines such as IFN‐β, IL‐12p70, IL‐6, IL‐10, and TNF‐α, within macrophages.^37^ Additionally, *P. gingivalis* OMVs transport gingipains, leading to an enhancement in the coaggregation of various bacteria, including *Staphylococcus aureus* and other *Streptococcus* spp., on the tooth surface.^38^ This process further aids in the invasion or attachment of *T. forsythia* to host epithelial cells.^39^

OMVs from other periodontal bacteria can also potentiate pathogencity by transmitting virulence factors. For instance, OMVs from *Tannerella forsythia* induce higher levels of monocyte chemoattractant protein‐1 (MCP‐1), IL‐8 and IL‐6 in PDLSCs and higher TNF‐α and IL‐8 expressions in macrophages when compared to their parent cells.^40^ Similarly, *Aggregatibacter actinomycetemcomitans* derived OMVs can transfer bioactive virulence factors (cytolethal‐distending toxins‐CDTs, outer membrane protein A‐OmpA) to human gingival fibroblasts, thereby influencing the occurrence and progression of periodontal disease.^41^ In mice, *Fusobacterium nucleatum* OMVs can induce a shift in macrophage phenotype from M0 to M1, ultimately contributing to periodontal tissue loss in mice.^42^ In addition, Gram‐positive periodontal bacteria *Filifactor alocis* OMVs may contribute to systemic bone loss in vivo through Toll‐like receptor 2.^43^

Periodontopathic bacteria and their byproducts can also enter the systemic circulation through periodontal tissue damage, potentially affecting distant sites.^44^ Recent research indicates that OMVs released by periodontopathogens contribute to the development of joint dysfunctions, brain disorders and various other systemic health conditions, aligning with the concept of ‘periodontal medicine’ and the known association between periodontal and systemic disease.^22^, ^45^, ^46^, ^47^ The conserved cargo within bacterial OMVs presents itself as an ideal biomarker, facilitating the rapid identification and differentiation of bacterial species.^48^

Recent findings suggest that OMVs from saliva biofilm of periodontally healthy indivudals reduce pro‐inflammatory cytokine production in oral keratinocytes, implying a role in periodontitis pathogensis by promoting plaque formation and inflammation.^49^ BEVs from periodontal pathogens may serve as potential biomarkers for periodontal disease detection, although the exact reasons for their pathogenic effects remain unclear. The role of OMVs from commensal bacteria or plaque needs further exploration.

However, none of the previously mentioned studies explored the cargo content of BEVs during their modulation of the immune response in host cells. The subsequent subsection delves into how microRNAs within BEVs might play a role in this immune response.

### 2.3 The role of periodontal bacterial EVs msRNAs in periodontitis

Within host EVs, particularly small EVs or exosomes, there is an abundance of extracellular RNAs (exRNAs), including mRNAs, microRNAs and other small RNAs. These exRNAs are encapsulated within EVs, offering protection from enzymatic degradation within biofluids. Therefore, it is plausible to speculate that bacterial OMVs may exist in bodily fluids, transporting bacterial cargos (ie. RNAs) that are shielded from enzymatic degradation. One current research has demonstrated the capability of *Escherichia coli* OMVs to traverse extensive distances throughout the body, reaching organs such as the heart, liver, kidney, spleen, and brain.^50^ This widespread distribution highlights the potentially far‐reaching impact of bacterial RNA delivery via OMVs. Notably, certain bodily fluids, including saliva and blood or GCF, may exhibit elevated concentrations of bacterial OMVs or BEVs from the local bacterial community within the oral cavity.^11^ Given the limited number of studies dedicated to investigating the RNA composition of BEVs from periodontal bacteria, there is an unexplored potential to understand the involvement of bacterial small RNAs (sRNAs) within these vesicles in host‐pathogen interactions.

Bacterial RNAs, including small non‐coding RNAs (ncRNAs) enriched within BEVs, may modulate bacteria‐to‐human interactions^51^, ^52^, ^53^ by regulating host immune response and cytokine secretion. Blenkiron et al. identified over 50 ncRNAs in the membrane vesicles of uropathogenic *E. coli* strain 536 and these ncRNAs were found to be delivered to bladder carcinoma cells.^54^ In subgingival plaque, a new class of bacterial small RNAs like miRNAs (namely msRNA) were found in OMVs secreted by periodontal pathogens, *A. actinomycetemcomitans*, *P. gingivalis*, and *T. denticola*.^55^ These msRNAs were P.G_45033, P.G_4378, P.G_122, P.G_16418, and P.G_25037 from *P. gingivalis* OMVs. Interestingly, these OMVs‐msRNAs were observed to transport RNAs to eukaryotic cells to suppress the expression of cytokines IL–5, IL‐13, and IL‐15 in Jurkat T cells. Additionally, OMVs exRNA derived from the periodontal pathogens *Actinomyces aggrebacter* and *A. actinomycetemcomitans* can traverse the blood–brain barrier (BBB), promoting the release of proinflammatory cytokine TNF‐α in the mouse brain.^46^, ^56^ It was speculated that OMVs‐msRNAs from periodontal pathogens might play a role in deactivating anti‐inflammatory cytokines, such as IL‐5 and IL‐13, or in evading the host adaptive immune response, warranting further investigation. Understanding the exact role and functions of msRNAs within OMVs of *P. gingivalis* and other periodontal pathogens, as well as expression profiling of disease associated bacterial small RNAs in vivo, remains an unexplored area.

In the subsequent section, we delve into the critical role of host immune cell‐derived EVs in investigating the pathogenesis of periodontitis.

### 2.4 Host immune cell derived EVs in the pathogenesis of periodontitis

Despite an expansion of knowledge in recent decades, particularly in recognising inflammation's role and host susceptibility, the precise mechanism of periodontitis pathogenesis remains elusive.^23^ What is established is the chronic inflammatory nature of the disease and involvement of immune cells, such as dendritic cells, macrophages, monocytes, and neutrophils. Among those immune cells, polarisation of M0 macrophage into M1 pro‐inflammatory or M2‐ anti‐inflammatory phenotypes corresponds to the relative differences in pro‐ and anti‐inflammatory activities during periodontitis.^57^ In a recent murine investigation, researchers observed the accumulation of M1/M2 derived small extracellular vesicles (sEVs) in periodontal tissues after intravenous injection of these sEVs. Notably, M1‐sEVs aggravated alveolar bone loss in ligature‐induced periodontitis in ovariectomised (OVX) mice due to elevated levels of miR‐30‐5p. Conversely, M2‐sEVs exhibited an opposing effect, partially mitigating periodontitis and fostering osteogenesis.^58^

In the periodontium, bacterial invasion into immune cells can lead to senescence of normal bystander dendritic cells by secreting inflammatory EVs. For example, Elsayed et al. found that *P. gingivalis* induces autocrine senescence in young dendritic cells through direct cellular invasion and amplifies senescence, in a paracrine fashion, of bystander DCs through the secretion of inflammatory dendritic cell EVs.^59^ The same group also demonstrated that EVs from *P. gingivalis*‐invaded dendritic cells lead to alveolar bone loss in a ligature‐induced periodontitis mice model.^60^

Despite limited research, EVs from host immune cells, both in the presence and absence of bacterial invasion, may play crucial roles in influencing the host immune response and associated tissue damage during periodontitis development. Therefore, the specific pathogenic contribution of host immune cell EVs in periodontitis remains largely unclear, warranting further research to explore their roles in disease pathogenesis.

The studies mentioned above suggest that both microbial and host‐derived EVs play a role in the development of periodontitis. Consequently, it is crucial to investigate the role of EVs in oral biofluids for diagnosing periodontitis, which will be further explored in the upcoming section.

## 3 DIAGNOSTIC ROLE OF MICROBIAL AND HOST EVS IN SALIVA AND GCF

Oral biofluids such as saliva and gingival crevicular fluid (GCF) contain a rich array of molecules from both host and microbial cells, including inflammatory mediators, antibodies, cytokines, epigenetic markers, tissue breakdown products and EVs.^61^, ^62^, ^63^, ^64^, ^65^, ^66^ Salivary biomarkers offer insight into host‐level inflammatiory or disease response, while the gingival crevicular fluids (GCF) provide information at the site level. Although current studies primarily focus on host cell derived EVs, there has been a notable focus on EVs derived from bacteria,^13^, ^43^, ^67^ due to their potential in diagnosing and predicting the progression of periodontitis.

### 3.1 Microbial EVs in periodontitis diagnosis

Research on isolating OMVs or BEVs from oral biofluids is limited. Lipopolysaccharide (LPS) is an ubiquitous constituent of OMVs, although there may be variations in LPS composition compared to the bacterial outer membrane itself. Studies have indicated that *P. gingivalis* OMVs exhibit distinctive features in their LPS content, characterised by extended sugar chains and deacylated lipid A, diverging from the composition observed in the bacterial cell wall.^68^ Notably, one study found elevated levels of LPS+ outer membrane vesicles and the release of OMVs from specific periodontal pathogens (*T. denticola*, *Eikenella corrodens*, *P. gingivalis*, and *F. nucleatum*) in salivary sEVs derived from periodontitis, compared to healthy controls.^69^

Since OMVs lack distinctive surface markers, isolating them directly from whole saliva or GCF is challenging. Our suggestion is to use an immunoaffinity method to eliminate host EVs and subsequently concentrate the remaining microbial EVs. This approach leverages the effectiveness of EXO‐NET immunobeads in substantially reducing microbial contamination in isolated saliva EV samples.^70^ Therefore, further exploration of OMV components, particularly surface markers, is essential for identifying potential OMV isolation methods.

### 3.2 Host derived EVs in periodontitis diagnosis

Relatively few studies have investigated host EVs and their cargo from saliva and GCF from a diagnostic perspective, but current findings suggest they could serve as diagnostic markers for periodontitis. It is well recognised that they contain host‐derived molecules including tetraspanins (CD9, CD 81 and CD63), lipids, integrins, growth factors and are enriched on a gene level with microRNA (miRNA), DNA, mRNA and protein that can provide disease‐specific diagnostic signatures.^12^, ^71^, ^72^, ^73^ Indeed, a pilot study demonstrated changes in 3 salivary sEV miRNAs in periodontitis patients when compared with healthy controls.^74^ Host sEVs related cytokines (IL6 and IL‐8)^70^ and mRNAs (*TNF‐α* and *OSX*)^73^ were increased in periodontitis compared to healthy controls. Protein analysis of saliva sEVs has also shown that innate immune response proteins were enriched in severe periodontitis patients, particularly for the complement component C6.^75^ Hence, host‐EVs in biofluids hold promise as biomarkers for the diagnosis of periodontal disease, overcoming common hurdles associated with studying biomarkers such as protein stability and contamination. However, challenges such as age matching among participants (ageing being a confounding factor to EV properties and circulation), standardised saliva collection, and heterogenous EV characterisation and standardisation need addressin in future research.^11^, ^76^

It is worth noting that none of the current research explored the origins of those host EVs and the uncertainty about whether they are secreted by immune cells remains unaddressed. As a result, we recommend that future research explores the origins of host EVs present in saliva and GCF using commercially available kits, for example, human MACSPlex EV Kit IO kit from Miltenyi Biotec enables the detection of 37 EV surface epitopes.

From the above sections, it is obvious that both microbial and host‐derived EVs can play a role in the development of periodontitis. Consequently, capturing the cargos carried by these EVs present in saliva and GCF could potentially serve as diagnostic biomarkers, reflecting the periodontal health status. In the subsequent section, we delve into their therapeutic role during periodontal treatment.

## 4 MICROBIAL BEVS AND HOST CELL DERIVED EVS FOR PERIODONTAL THERAPY

The therapeutic potential of host cell derived EVs lies in their ability to facilitate interceullar communication, modulate immune responses in surrounding environments, exhibit low immunogenicity and serve as drug delivery vehicles.^77^ Since EVs have similar characteristics to their parent cells, they present with low toxicity and immunogenicity. They remain stable in circulation and can efficiently traverse biological barriers, thereby enhancing the bioavailability of the molecules they carry.^78^ Furthermore, EV surfaces can be engineered to improve their targeting specificity, immunogenicity, biodistribution and pharmacokinetics.^79^

In the context of periodontal regeneration, immune cell‐derived EVs, akin to their parent cells, may stimulate the recruitment of local periodontal MSCs, promote osteogenesis, and modulate the inflammatory environment to favour tissue regeneration. In this section, we provide a summary of the impact of periodontal mesenchymal cell‐derived EVs, followed by an exploration of the potential role of immune cell‐derived EVs in periodontal regeneration.

### 4.1 Microbial BEVs as potential periodontal therapy

While the therapeutic potential of host‐EVs from periodontal cells or immune cells is evident in periodontal care, the exploration of BEVs from periodontal bacteria is still in its early phase. This is because all OMVs contain endotoxin, specifically lipopolysaccharide (LPS), which poses a significant concern in considering OMVs for therapeutic applications. However, the cost‐effectiveness of large‐scale BEV production makes it promising for potential periodontal treatment in terms of antibiotic drug delivery vehicles or vaccine development. For instance, *E. coli* OMVs coated with antibiotics (rifampicin) showed superior antibacterial efficiency compared to conventional antibiotics treatment.^80^ Thus, it is reasonable to speculate that periodontal bacterial BEVs can be employed for antibiotic drug delivery to enhance the uptake of antibiotics.

Studies have indicated that *P. gingivalis* OMVs maintain the immunodominant determinant of the bacterium, leading to increased production of salivary IgA, serum IgG, and IgA in mice upon intranasal administration.^81^ The robust immunogenicity of *P. gingivalis* OMVs primarily arises from LPS, and the immunoreactivity diminishes significantly upon serum absorption with LPS.^82^ Despite these advancements, the utilisation of OMVs for vaccination is still in its early stages, demanding extensive efforts to mitigate potential side effects.

Despite the promising aspects of low cost and large‐scale production associated with biofilm‐derived extracellular vesicles (BEVs), their potential for periodontitis treatment is still largely unexplored. This is primarily due to significant challenges related to high toxicity and the isolation and characterisation of BEVs.

### 4.2 Host periodontal mesenchymal cell derived EVs in periodontal therapy

EVs originating from mesenchymal stromal cells, whether odontogenic or non‐odontogenic, have been shown to mitigate oxidative damage and modulate the micro‐environment's immune response, creating a less inflammatory and more favourable environment for tissue repair and regeneration.^83^, ^84^ At a cellular level, MSC‐derived EVs have been shown to enhance the activities of cells and processes crucial for regeneration, for example, osteogenesis, angiogenesis and the proliferation, differentiation and migration of fibroblasts and osteoblasts.^85^, ^86^ Furthermore, increasing evidence suggests that periodontal ligament stem cell EVs (PDLSCs‐EVs) from cells pre‐stimulated with mild inflammatory cytokines can promote osteogenic differentiation of PDLSCs.^87^ In a recent study, small extracellular vesicles (sEVs) obtained from three distinct periodontal cells (PDLCs, gingival fibroblasts, and osteoblasts) demonstrated increased in vitro osteogenic differentiation in buccal fat pad MSCs when compared to two other subtypes of EVs, namely microvesicles and apoptotic bodies.^17^ Another study demonstrated that human gingival fibroblast derived microvesicles (hGFs‐MVs) can reduce pro‐inflammatory cytokines when human gingival keratinocytes (OKF6/TERT2 cell line) cultured on GF‐MVs loaded nano‐engineered titanium surfaces.^88^ These selected studies demonstrated that EVs from dental mesenchymal stem cells show promising potential in periodontal regeneration. In comparison, despite the known roles of immune cells in regenerative and reparative processes, there is limited literature on immune cell derived EVs.

### 4.3 Host immune cell derived EVs in periodontal therapy and repair

Immune cell derived EVs, especially macrophage EVs, have been explored in osteogenesis in various aspects. In a rat calvaria model, macrophage‐derived EVs were shown to influence the differentiation of mesenchymal stem cells and osteoblast function during bone regeneration.^89^ This model demonstrated polarisation‐specific control of bone regeneration by macrophage EVs, with naïve (M0) and M2 EVs promoting repair, while M1 EVs seem to inhibit this process. This is substantiated by other studies where macrophage EVs have been shown to regulate macrophage polarisation and suppression of inflammation through miRNAs such as miR‐99a/146b/278a and miR‐223.^90^, ^91^ Similarly, cementoblasts have been affected by the introduction of macrophage EVs with M2 EVs promoting cementoblast mineralisation, albeit in an orthodontic tooth movement model.^92^ Although these results demonstrate potential therapeutic applications for immune cell derived EVs, a key limitation is that they are not applied within a periodontitis model.

Herein, we summarise selected studies exploring the role of immune cell derived EVs as periodontal therapeutic agents during inflammation and bone regenerative medicine^58^, ^93^, ^94^, ^95^, ^96^ (Table 1).

**TABLE 1:** Selected studies using immune cell derived EVs in periodontal regenerative therapies.

Authors	Parent and recipient cells	EV isolation	EV characterisation	Main findings
Cui et al., 2023^93^	The RAW 264.7 macrophage cell line derived sEVs in primary LPS‐induced hPDLCs	The conditioned medium was ultrafiltered through MWCO of 200 kDa. Centrifuged at 1000 *g* for 30 min, 10 000 *g* for 30 min. Ultracentrifuged at 100 000 *g* for 90 min to pellet exosomes and ultracentrifuged at 100 000 *g* for 60 min to wash away impurities.	BCA assay TEM Western blot NTA Size: 118.15 ± 0.989 nm	In vitro: Engineered M2 macrophage‐derived exosomes accelerate osteogenic and cementum differentiation of hPDLCs under inflammatory conditions and mediate immune reprogramming and polarisation of macrophages from pro‐inflammatory to anti‐inflammatory phenotypes. In vivo: Injection of GelMA hydrogel combined with melatonin‐engineered M2 macrophage‐derived exosomes reverses alveolar bone loss in a rat model of ligation‐induced periodontitis.
Li et al., 2022^58^	M1 and M2 macrophage derived sEVs in mesenchymal stem cells (MSCs)	Centrifuged at 500 *g* for 10 min, 10 000 *g* for 20 min, and 120 000 *g* for 60 min. The isolated M1‐sEVs and M2‐sEVs were resuspended in PBS and centrifugated at 120000 *g* for 30 min.	TEM NTA Western blotting Size: 30–150 nm	In vitro: M1‐sEVs significantly inhibited the osteogenesis of MSCs, while M2‐sEVs promoted the formation of mineralised nodules of MSCs. In vivo: M1‐sEVs inhibit osteogenesis, while M2‐sEVs promote osteogenesis of MSCs in ligature‐induced periodontitis models.
Liao et al., 2022^94^	M0, M1 and M2 macrophages EVs in hPDLSCs	Centrifuged at 300 *g* for 10 min, 2000 *g* for 10 min, 10 000 *g* for 30 min with 4°C. Ultracentrifuged at 100 000 *g* for 90 min with 4°C to precipitate exosomes.	TEM Western blot EXOCET Exosome Quantitation Assay Kit Size: 50–150 nm	M2‐exo augmented mineralised nodule formation and upregulated ALP and OCN expression in hPDLSCs, while M0‐exo had no significant effect. Compared to M0‐exo, the expression of hsa‐miR‐6085, hsa‐miR‐4800‐5p, hsa‐miR‐4778‐5p, hsa‐miR‐6780b‐5p and hsa‐miR‐1227‐5p was significantly up‐regulated in M2‐exo compared to M0‐exo.
Chen et al., 2022^95^	Reparative M2‐like macrophages EVs in BMSCs	Centrifuged at 3000 *g* for 15 min, followed by filtration with a 0.45 μm filter. Ultracentrifugation for 90 min at 120 000 *g* at 4°C. The precipitate was resuspended by PBS and spun for an additional 90 min at 120 000 *g*.	TEM NLS Western blot Size: 30–150 nm	In vitro: The expressions of Alp, Col1a, RUNX2, and Ocn were significantly upregulated in the M2D‐Exos group in a concentration‐dependent manner. M2‐Exos exerts osteoprotective effects by regulating the differentiation of bone marrow stromal cells (BMSCs) and bone marrow‐derived macrophage (BMDM). In vivo: Alveolar bone resorption was significantly reduced in M2‐Exos‐treated mice compared with the control group, and the bone protective effect of the buccal alveolar bone was stronger than that of the palatal side. M2‐Exos‐derived IL‐10 reduces periodontitis in mice alveolar bone resorption.
Elashiry et al., 2020^96^	Dendritic cells from tibia and femur bone marrow from 6‐ to 8‐week‐old mice in CD4+ T cells	Centrifugations at 500 *g* for 5 min, 2000 *g* for 20 min, 10 000 *g* for 30 min. Ultrafiltration 3× with 0.2 μm and 3× with 100 kDA filters and ultracentrifugation for 1.5 h at 120 000 *g*. EXO pellets were washed with PBS and ultra‐centrifuged 2× at 120 000 *g* for 1.5 h.	Western blotting TEM NTA Size: 30–150 nm	In vitro: RegDC EXO induces TGFβ1 and IL10‐mediated immunomodulatory effects on recipient DCs. RegDC EXO increases the resistance of recipient DCs to LPS‐mediated maturation and reduces antigen presentation ability. RegDC EXO is also taken up by CD4 T cells inhibited in vitro proliferation and promote TGFβ1‐mediated Tregs induction. In vivo: RegDC EXO effectively inhibits inflammatory alveolar bone loss. TGF‐β and IL‐10‐loaded regDC EXOs reprogram immune responses and suppress inflammatory bone loss.

Abbreviations: Alp, alkaline phosphatase; BMP2, bone morphogenetic protein 2; Col11a, collagen type 1 alpha 1; EXO, exosomes; FLS, fibroblast‐like synoviocyte; GelMA, Gelatin methacryloyl; hPDLSCs, human periodontal ligament stem cells; IL10, interleukin 10; IMC, intrafibrillarly mineralised collagen; IMC‐sEVs, small extracellular vesicles derived from intrafibrillarly mineralised collagen; IRS‐1, insulin receptor substrate 1; LPS, lipopolysaccharide; M0‐exo, M0 macrophage‐derived exosomes; M1‐exo, M1 macrophage‐derived exosomes; M2, M2 macrophage; M2D‐Exos, M2 macrophage‐derived exosomes; M2‐exo. M2 macrophage‐derived exosomes; MMP13, matric metalloprotease 13; NEs‐Exo, neutrophil‐derived exosomes; Ocn, osteocalcin; RA, rheumatoid arthritis; RegDC, regulatory dendritic cell; RUNX2, runt‐related transcription factor 2; TGFβ1, transforming growth factor beta 1; Th17, T helper type 17; TNF‐α, tumor necrosis factor alpha; Treg, T regulatory cell; uPB‐Exo, ultrasmall Prussian blue nanoparticle exosomes.

**FIGURE 2:**
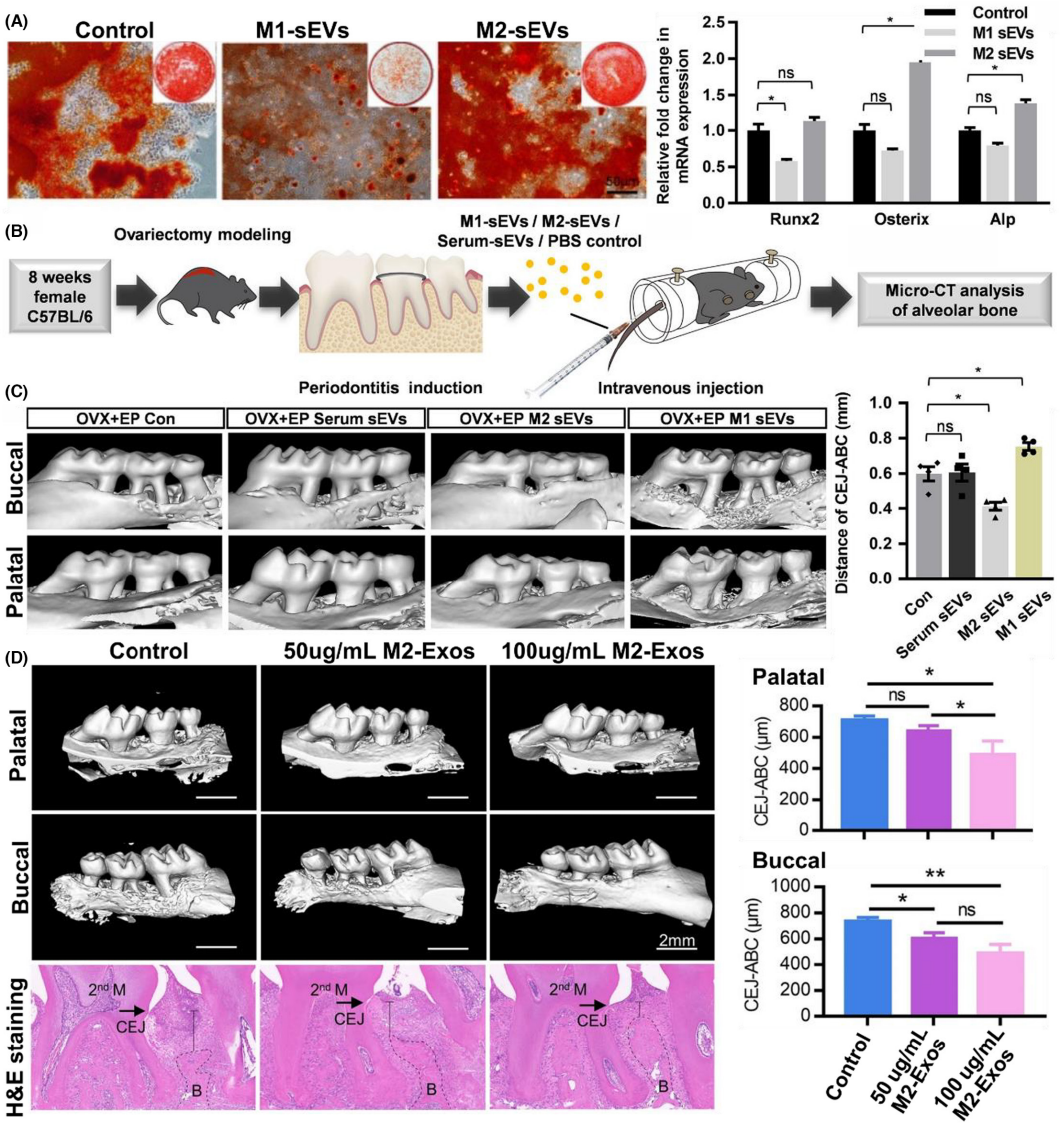
The impact of M1 or M2 derived EVs in a murine model of periodontitis. (A) Alizarin red staining of cultured MSCs cultured in control, M1 or M2 EVs showing increased osteogenic activity of MSCs cultured in M2‐EVs and increased expression of osteogenic genes including Runx2, Osterix and ALP. (B) Schematic model demonstrating the intravenous injection of macrophage EVs following periodontitis induction. (C) Micro‐CT images demonstrating control, reduced bone loss in mice injected with serum M2 EVs versus increased bone loss when injected with M1 EVs − adopted from Li et al.^58^ (D) Micro‐CT and accompanying histology showing a reduced bone loss in experimental periodontitis sites injected with M2 Exos adapted from Chen et al.^95^ Alp, Alkaline phosphatase; LPS, lipopolysaccharide; M1 sEVs, M1 macrophage derived small extracellular vesicles; M1, M1 macrophages; M1‐exo, M1 macrophage‐derived exosomes; M2 sEVs, M2 macrophage derived small extracellular vesicles; M2, M2 macrophage, MSC, mesenchymal stem cells; M2, M2 macrophage; M2‐Exos, M2 macrophage‐derived exosomes; OVX, ovariectomy; RUNX2, runt‐related transcription factor 2.

Given that periodontitis is a chronic inflammatory condition involving both the innate and adaptive immune response, altering cellular behaviour and differentiation through immune cell derived EVs could be a viable therapeutic approach for combating bone loss due to periodontitis. In a murine model with experimental periodontitis, M1 and M2 derived EVs were shown to inhibit and promote osteogenesis respectively both in vitro and in vivo (Figure 2A–C).^58^ In vitro, M1 EVs significantly inhibited the osteogenesis of MSCs while M2 EVs promoted the formation of mineralised nodules of MSCs. In vivo, alveolar bone loss was attenuated in mice intravenously injected with EVs pre‐loaded with an inhibitor of miR‐303‐5p, a miR highly expressed in M1 macrophages. Similar results are also reported by other authors examining macrophage derived EVs.^93^, ^95^ Chen et al. highlighted that M2‐EVs could promote the osteogenesis of BMMSCs via the delivery of multiple miRNAs and through the IL‐10/IL‐10R signalling pathway, resulting in reduced bone resorption in mice within a ligature‐induced periodontitis model^95^ (Figure 2D).

Similarly, M2‐derived EVs loaded with melatonin were demonstrated by Cui et al. to increase the osteogenic and cementogenic potential of human periodontal ligament cells (hPDLSCs) under inflammatory conditions. Importantly, these loaded EVs were also shown to drive an appropriate and timely reprogramming of macrophages from an M1 to M2 phenotype, producing reduced inflammation and accelerated periodontal healing.^93^ Although the exact mechanism of how M2 EVs can promote the osteogenic differentiation of human PDLSCs is unknown, it is postulated that miRNAs such as hsa‐miR‐6085, hsa‐miR‐4800‐5p, hsa‐miR‐4778‐5p, hsa‐miR‐6780b‐5p and hsa‐miR‐1227‐5p have a significant role.^94^

**FIGURE 3:**
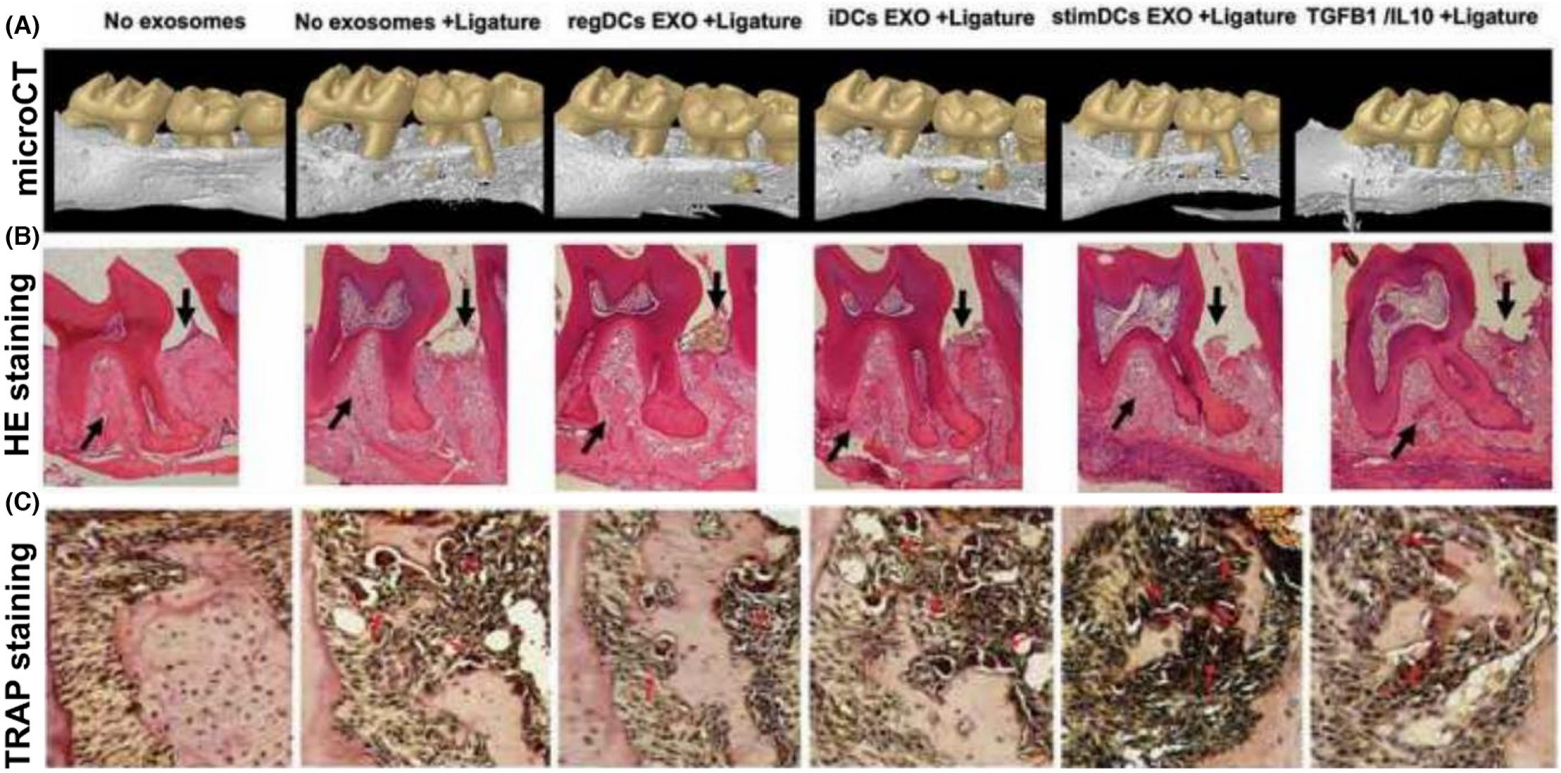
Compilation of micro‐CT, HE staining and TRAP staining demonstrating the effect of various subtypes of dendritic cell exosomes when injected into experimental periodontitis sites in mice. regDCexo reduced alveolar bone loss while stimDC exo increased bone loss with iDC Exo and free TGFβ/IL‐10 showing limited inhibitory effects against inflammatory bone loss. This was adopted from Elashiry et al.^96^ iDC Exo, immune immature dendritic cell exosomes; IL‐10, interleukin‐10; regDC EXO, immune regulatory dendritic cells exosomes; stimDCs Exo, immune stimulatory dendritic cell exosomes; TGFB1, Tissue growth factor 1; TRAP, Tartrate resistant acid phosphatase.

The concept of utilising EVs as carriers of therapeutic cargo in periodontal therapy has also been demonstrated for dendritic cells. Preliminary studies examining DC‐derived EVs in an experimental periodontitis model have shown the ability of DC EVs enriched with TGF β/IL‐10 to reduce alveolar bone loss. In mice, DC EVs enriched with TGF β/IL‐10 displayed a high infinity for inflamed sites when delivered via an IV route or locally into the soft tissues overlying the alveolar bone. These EVs suppressed the induction of Th17 effectors while promoting T regulatory cell recruitment, resulting in the inhibition of bone resorptive cytokines and reduction in osteoclastic bone loss^96^ (Figure 3). Consistent with the current understanding of the role inflammation has in periodontal destruction, it is not surprising that sites exposed to immunostimulatory (stimDCs) exos resulted in the greatest amount of bone loss. Additionally, this study demonstrated the ability and importance of exosomes to protect encapsulated immunoregulatory cargo, in this case TGFβ1 and IL‐10, with free TGFβ1/IL‐10 failing to prevent bone loss.

Immune cell derived EVs could also be used to the modification and engineering of biomaterials in periodontal therapy. Recently, EVs from intrafibrillarly mineralised collagen (IMC) treated macrophages facilitated MSC osteogenesis through the BMP2/Smad5 pathway.^97^ Interestingly, blocking sEVs secretion significantly impaired the proliferative, immunonomodulative and osteogenic effects of the MSCs, implicating the potential of macrophage‐EVs as a functional tool in biomaterial‐mediated bone regeneration.^97^

Current investigations predominantly focus on the utilisation of dental mesenchymal stem cell‐derived EVs for the treatment of periodontitis, while there is a paucity of studies exploring the potential of immune cell‐derived EVs. Among the five studies included, all relied on cell lines as the EVs source material, suggesting that primary cells might offer a more dependable source for isolating EVs. However, primary cell cultures present their own set of challenges, including difficulties in attaining sufficient cell numbers and alterations in morphology and phenotype following in vitro cultivation. Maintaining cell surface markers is imperative to ensure the fidelity of the cell type. Furthermore, all five studies employed ultracentrifugation (UC) for EV isolation from immune cells, a process known for its time‐intensive nature and the potential for protein or EV aggregation.^98^, ^99^, ^100^ There is a pressing need for further exploration of alternative isolation methods to enhance EV yield and purity.

In four out of five studies,^58^, ^93^, ^95^, ^96^ an in vivo ligature‐induced periodontitis model was employed to assess the impact of immune cell EVs. However, this model does not directly correlate with clinical periodontitis in humans. Moreover, the dosage, frequency, and method of delivery of EVs play a crucial role in achieving optimal therapeutic outcomes in vivo. In one study, local delivery involved administering either 50 μg/mL or 100 μg/mL of M2‐Exo for durations of 1, 4, and 7 days,^95^ while another study utilised the delivery of 10^8^ particles of DCs EVs for 2 days post‐ligature removal.^96^ Additionally, one investigation opted for intravenous injection, delivering 100 μg in 200 μL of PBS every week for 4 weeks following periodontitis induction.^58^ Notably, one study^95^ employed injectable gelatin methacrylate (GelMA) as a vehicle for engineered M2‐EVs in a rat model of ligature‐induced periodontitis. Exploration into more biomaterials (i.e., 3D bioprinting^101^) as EVs delivery vehicles is signficant. These variations in EV delivery and concentration create limitations in the interpretation of the degree of effect.

These investigations consistently demonstrated enhanced bone regeneration resulting from M2‐EVs and DC‐EVs. However, the aspect of soft tissue regeneration was overlooked in these studies. Future research endeavours may benefit from addressing both periodontal soft and hard tissue repair following the administration of immune cell EVs. It is essential to recognize that the dosage, frequency, and delivery methods varied across these four in vivo studies. Hence, optimising EV dosage, frequency, and delivery systems warrants consideration in future research on in vivo EV applications.

## 5 SUMMARY AND KEY CONSIDERATIONS FOR EV RESEARCH IN PERIODONTOLOGY

### 5.1 Summary of microbial‐ and host immune cell‐EVs in periodontitis

**FIGURE 4:**
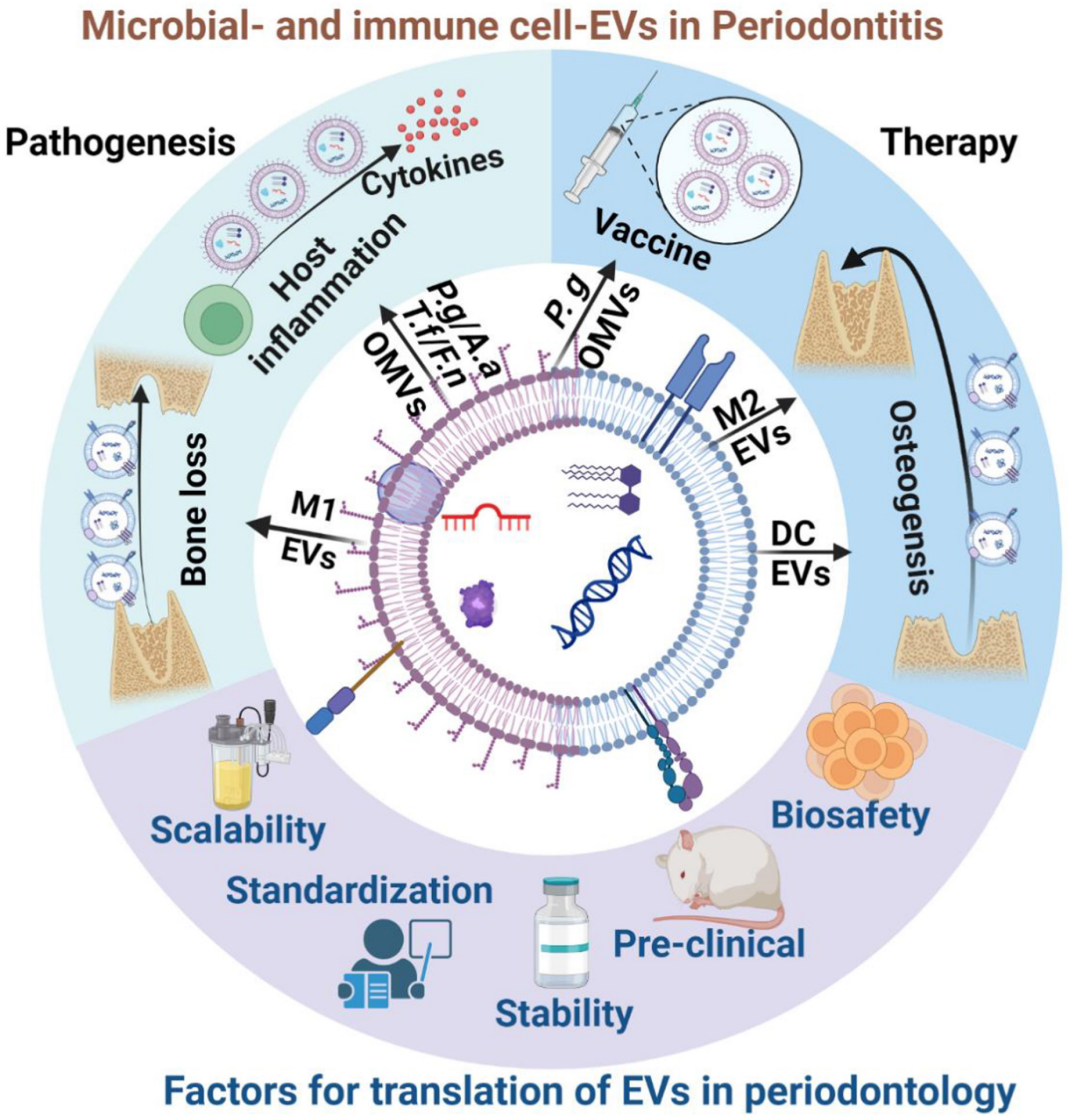
Microbial and immune cell derived EVs in periodontitis and key factors for translation of EVs in periodontology. OMVs derived from periodontal pathogens, such as *A.a*., *P.g*. and *T.f*., can trigger excessive inflammatory responses in the host leading to destruction. Similarly, M1‐EVs have been shown to enhance bone loss. EVs originating from immune cells can also be potential targets for therapy, with M2‐EVs and DC‐EVs being shown to promote osteogenesis and reduce inflammatory bone loss. Bacterial OMVs such as those from *P. gingivalis* can be used as target antigens for vaccine therapies. The clinical translation of EVs encounters hurdles such as biosafety concerns, yield variability, standardisation issues, and stability and scaling challenges. *Aa*, *Aggregatibacter actinomycetemcomitans*; DC, dendritic cell; *Fn*, *Fusobacterium nucleantum*; M1, M1 macrophage; M2, M2 macrophage; OMV, outer membrane vesicles; *Pg*, *Porphyromonas gingivalis*; *Tf*, *Tannerella forsythia*.

Research is scarce on the utilisation of EVs derived from microbial and host immune cells for understanding periodontitis pathogenesis, diagnosis, and treatment. Available studies suggest that these EVs may play dual roles, with both positive and negative effects on periodontal disease pathogenesis and therapeutic approaches (Figure 4). Current research suggests that various periodontal pathogens (*T. forsythia*, *A. actinomycetemcomitans*, *F. alocis*, *F. nucleatum*) derived microbial EVs can potentially initiate host inflammation by elevating inflammatory cytokines in various host immune cells and periodontal mesenchymal cells.^37^, ^40^, ^41^, ^42^, ^43^ Additionally, *P. gingivalis* OMVs can potentially increase the local biofilm formation on tooth surfaces.^38^ Furthermore, immune cell M1‐EVs can lead to alveolar bone loss in animal models of periodontitis with and without osteoporosis.^58^ In terms of periodontal therapy, *P. gingivalis* OMVs have been shown to be potential vaccine candidates due to their capability of initiating immune response in animals. Both M2‐EVs and DC‐EVs are shown to promote bone regeneration in ligature‐induced periodontitis disease animal models.^58^, ^95^, ^96^

At present, there is a paucity of data from clinical studies on the therapeutic potential of microbial‐ and host‐derived EVs in periodontitis. The regenerative potential of EVs derived from autogenous adipose stem cells in periodontitis treatment is currently being investigated in an ongoing clinical trial (NCT04270006) with stage III/IV periodontitis patients. In this trial, the EVs are administered locally via injection into periodontal pockets as an adjunct to conventional scaling and root planing. At present, no outcomes or findings from this trial have been disclosed. There are no current human clinical trials utilising EVs derived from immune cells or periodontal pathogens despite the relevance of these EVs in both disease pathogenesis and reparative processes.

Although bacterial OMVs and host derived EVs have the capability to act as diagnostic determinants for disease activity, a significant gap remains in translating this to clinical diagnostic tools. One of the main challenges is distinguishing bacterial EV from host derived EVs in body fluids. Nevertheless, information derived from EV studies continues to contribute to the understanding of the periodontitis disease process.

In the following subsection, we outline several critical limitations associated with EV research in periodontology. These limitations may serve as guidelines for future researchers in the field.

### 5.2 Limitations of EV research in periodontal diagnosis and therapy

Aside from scarcity of research data, key limitations in the periodontal EV literature are commonly applicable to EV research in general, such as heterogeneity in methods of EV extraction and characterisation, and isolation from non‐target sites. This is despite the availability of published guidelines for EV research.^28^ Indeed, in most studies, immune cell EVs have been derived from cell lines rather than sites from the periodontium, highlighting the need for consistent methods and evaluation of EVs from actual periodontal sites in future research. Challenges in EV research and translation to clinical applications include the following:

- *Heterogeneity* − EVs are heterogeneous populations with variations in size, cargo, membrane composition, biogenesis, and importantly biological function. Currently, the absence of standardised isolation and characterisation techniques for EVs and the diversity and variability in the number of EVs produced by different methods render achieving consistency, standardisation, and reproducibility a challenge.^8^, ^16^, ^102^ This heterogeneity can affect the interpretation of data from EV research, including the understanding of EV cargos, functions and therapeutic efficacy.
- *Cargo loading and delivery* − Efficiently loading therapeutic cargo into EVs and ensuring controlled release at target sites presents challenges. Optimisation of methods for loading specific molecules is needed. For EVs to have sufficient therapeutic efficacy in periodontal treatments, the homing and uptake of EVs to specific sites and targets is necessary. We have proposed a novel delivery method for EVs by using 3D bioprinting of EVs, which is further supported by promising outcomes reported by using ‘cell‐free’ periodontal tissue regeneration approaches.^101^, ^103^, ^104^ The targeted EV delivery is particularly required in the case of regenerative periodontal therapy, where successful outcomes are not only dependent upon coordinated interactions between various tissue types. Targeted delivery may be an issue in EV based therapies, as they can be uptaken by different cell types.
- *Bioavailability* − Administered EVs may encounter obstacles in reaching target tissues, especially from systemic EV delivery. EVs may be cleared by the immune response and degraded by enzymes within 10 minutes of entering the bloodstream,^105^ thereby diminishing their bioavailability and in vivo stability.
- *Scalability* − For any therapeutic modality efficient and cost‐effective production is required to meet demands of clinical applications. Developing consistent, reproducible methods for producing clinical‐grade EVs is also currently challenging. Presently, the majority of immune cell or bacteria cultures are conducted using 2D static flasks or well‐plates. To enhance the production of EVs at a larger scale, it is recommended to utilize 3D cell culture systems, such as 3D melt electrowritten polycaprolactone scaffolds,^106^ or to implement dynamic bioreactor systems.^107^
- *Ethical and consent issues* − The use of EVs for therapeutic purposes may involve obtaining and processing biological materials from donors, and ethical considerations, such as appropriate informed consent must be carefully addressed. Compliance with the latest MISV guidelines,^108^ as well as those mendated by regulatory agencies, ie US FDA and Australia's TGA, are required. *It should be noted*, however, that there is currently no universally accepted international directive specifically addressing the utilisation of EVs in clinical research.

Additionally, key factors for using microbial and host immune cell derived EVs for periodontitis diagnosis and treatment include the source of EVs, parental cell culture conditions and donour health status.

- *Cell source of EVs*: It is noteworthy that existing studies have exclusively employed immortalised cell lines for the extraction of host EVs. However, there are intrinsic differences between cell lines and human primary cells. Future investigations should prioritize the use of human primary cells to better mimic in vivo scenarios. In the context of microbial EVs, current research relies on the in vitro cultivation of single periodontal pathogen species to obtain BEVs. However, dental biofilm consist of multi‐microbial species in a complex three‐dimensional structure. Utilising BEVs from the in vitro culture of biofilms^49^ may provide a closer reflection to in vivo conditions. However, challenges remain in the similarity between the components of OMVs from in vitro cultured biofilm and OMVs from native oral biofilms.
- *Health status of donors*: The fundamental patient details essential for inclusion in donor‐related studies involve age, gender, systemic health and periodontal health, following the latest staging and grade guidelines.^109^ Specifically, in investigations focusing on periodontal diagnosis, the strong recommendation is to recruit participants who are matched in terms of age and gender when comparing patients with different periodontal disease status. Furthermore, adhering to these clinical reporting criteria is equally crucial for prospective studies involving the collection of host immune cells or dental plaque for EV isolation.

### 5.3 Future research perspectives

Further exploration of EVs derived from periodontal pathogens and other commensal bacteria is essenitial for further understanding of periodontitis pathogenesis and devising effective therapies. The complexity of the periodontal microenvironment extends beyond just periodontal pathogenic bacteria, necessitating research into the various EVs produced by the diverse bacteria present in dental oral biofilms. Unlike bacterial EVs from single species, those from oral biofilms can more accurately capture the intricate dynamics of the oral environment.

Moreover, there is a need for additional research to differentiate between host and microbial EVs when diagnosing periodontitis using oral biofluids. The role of innate immune cell derived EVs and their interaction with the host microbiome and cells requires further exploration. When studying host immune cell derived EVs in vitro, using primary cells is crucial despite the challenges they present, such as limited cell numbers and senescence post in vitro culture. While still in its infancy, the translation of these EVs from in vitro to in vivo pre‐clinical models holds promise for future clinical applications.

## 6 CONCLUSIONS

Although studies on the topic are still limited, current research supports a potential role of microbial and host immune cell derived EVs in the pathogenesis, diagnosis and treatment of periodontitis. Evidence suggests that OMVs from periodontal pathogens could serve as inflammatory triggers and potential targets for vaccination (i.e., *P. gingivalis* OMVs). Additionally, EVs from immune cells, particularly M2‐EVs, may promote in vivo bone regeneration, whereas M1‐EVs could excentuate bone loss during the pathogenesis of periodontitis. These initial findings contribute to a deeper understanding of the complexities of periodontitis, paving the way for the development of diagnostic, prognostic, and therapeutic approaches by leveraging the properties of microbial and host immune cell derived EVs.

## CONFLICT OF INTEREST STATEMENT

The authors declare no conflicts of interest.

## Data Availability

The data that support the findings of this study are available from the corresponding author upon reasonable request.
